# The HALLMOUNT Score: Development of a Novel Multidimensional Prognostic Model for Solid Tumors, with Initial Clinical Application in Grade 4 Adult-Type Diffuse Gliomas

**DOI:** 10.3390/medicina61122232

**Published:** 2025-12-17

**Authors:** Ahmet Unlu, Asim Armagan Aydin, Banu Ozturk, Cezmi Cagri Turk, Mustafa Yildiz

**Affiliations:** 1 Department of Clinical Oncology, University of Health Sciences, Antalya Education and Research Hospital, 07100 Antalya, Turkey; drbanutr@yahoo.com (B.O.); drmyildiz@yahoo.com (M.Y.); 2 A Department of Neurosurgery, University of Health Sciences, Antalya Education and Research Hospital, 07100 Antalya, Turkey; drcezmiturk@gmail.com

**Keywords:** HALLMOUNT, glioblastoma, biomarker, grade 4 adult-type diffuse glioma, prognosis, central nervous system, brain tumor, survival

## Abstract

*Background and Objectives:* Grade 4 adult-type diffuse gliomas remain the most aggressive primary central nervous system malignancies, with limited prognostic tools beyond molecular classification. This study introduces the HALLMOUNT score, a multidimensional prognostic index integrating hematologic, biochemical, and clinical parameters to capture the interplay between tumor biology and systemic host response. *Materials and Methods:* A total of 227 patients with histologically confirmed grade 4 adult-type diffuse glioma were retrospectively analyzed. The HALLMOUNT score incorporated nine pretreatment variables: hemoglobin, albumin, lactate dehydrogenase (LDH), lymphocyte, monocyte, Eastern Cooperative Oncology Group (ECOG) performance status, uric acid, neutrophil, and thrombocyte counts. Receiver operating characteristic (ROC) analyses determined optimal cut-offs, and Cox regression models evaluated prognostic performance for overall (OS) and progression-free survival (PFS). *Results:* High HALLMOUNT scores (≥2.5) were significantly associated with older age, comorbidities, poor ECOG status, isocitrate dehydrogenase (IDH)-wild phenotype, lower resection rates, and reduced treatment responses. ROC analysis showed predictive accuracy comparable to CAR and PIV (AUC = 0.650). High scores independently predicted inferior OS (HR = 2.78, *p* < 0.001) and PFS (HR = 2.76, *p* < 0.001). *Conclusions:* The HALLMOUNT score provides a simple, cost-effective, and biologically grounded biomarker reflecting both tumor aggressiveness and host vulnerability. It enables refined risk stratification, supports individualized therapeutic planning, and warrants prospective validation in molecularly defined and multicenter cohorts.

## 1. Introduction

According to the 2021 revision of the World Health Organization (WHO) classification of central nervous system (CNS) tumors [1], grade 4 adult-type diffuse gliomas comprise glioblastoma, isocitrate dehydrogenase (IDH)-wild-type, astrocytoma, IDH-mutant, and diffuse hemispheric glioma, *H3F3A G34R/V*-mutant. Collectively, these entities represent the most biologically aggressive forms of adult diffuse gliomas, distinguished by marked histopathological and molecular heterogeneity, infiltrative growth, and rapid proliferation, which confer resistance to standard therapies and drive early recurrence and high mortality [2,3,4]. The global incidence is approximately 5–7 cases per 100,000 individuals annually, with a higher prevalence in males and in those over 65 years of age [2,5]. The current standard of care of maximal safe surgical resection followed by radiotherapy and temozolomide-based chemotherapy extends survival modestly [6], with a median overall survival of 12–18 months and five-year survival rates below 10% [7,8]. Despite emerging approaches such as immune checkpoint inhibitors [9], oncolytic viruses [10], and dendritic-cell vaccines being promising in early phase trials [11], their clinical efficacy in routine practice remains inconclusive. Consequently, increasing attention has been paid to blood-based biomarkers and composite prognostic indices that could refine risk stratification and enhance prognostic accuracy by capturing both tumor-intrinsic biology and host systemic responses.

Systemic immune–inflammation plays a fundamental role in cancer pathogenesis by shaping tumor initiation, progression, and therapeutic resistance through chronic cytokine activation and immune dysregulation [12]. In aggressive CNS tumors, such as high-grade gliomas, this persistent inflammatory milieu drives angiogenesis, immune evasion, and metabolic reprogramming, ultimately leading to unfavorable clinical outcomes [13]. Neutrophils promote glioma progression by releasing pro-angiogenic cytokines, proteolytic enzymes, and reactive oxygen species, which stimulate endothelial proliferation, degrade extracellular matrices, and suppress antitumor immunity [14]. Conversely, lymphocytes, particularly cytotoxic T cells, are central to immune surveillance and tumor eradication; thus, lymphopenia indicates impaired adaptive immunity and weakened host response [15]. Under the influence of glioma-derived cytokines such as IL-6 and CCL2, they differentiate into tumor-associated macrophages (TAMs) that secrete transforming growth factor-β (TGF-β), vascular endothelial growth factor (VEGF), and matrix metalloproteinases (MMPs), fostering an immunosuppressive and pro-invasive microenvironment [16]. Platelets further support tumor progression by shielding circulating tumor cells and releasing VEGF and platelet-derived growth factor (PDGF), which enhances vascular remodeling [17]. Hypoalbuminemia reflects a chronic catabolic and inflammatory state, signifying systemic frailty and metabolic exhaustion [18]. These interconnected mechanisms form the biological basis of inflammation-based hematologic indices such as the neutrophil-to-lymphocyte ratio (NLR), platelet-to-lymphocyte ratio (PLR), monocyte-to-lymphocyte ratio (MLR), and C-reactive protein-to-albumin ratio (CAR), which integrate immune activation, inflammation, and nutritional status into quantifiable parameters. Numerous studies have demonstrated that elevated NLR, PLR, MLR, and CAR values are significantly associated with early recurrence, shorter overall survival (OS), IDH-wild phenotype, advanced age, and poor Eastern Cooperative Oncology Group (ECOG) performance status (PS) in patients with grade 4 adult-type diffuse gliomas [19,20,21,22], highlighting their prognostic relevance as indicators of systemic immune-inflammatory dysregulation.

In recent years, multidimensional inflammation- and nutrition-based indices, such as the systemic immune-inflammation index (SII) [23], systemic inflammation response index (SIRI) [24], pan-immune-inflammation value (PIV) [25], global immune-nutrition-inflammation (GINI) index [26], and hemoglobin–albumin–lymphocyte–platelet (HALP) score [27] have emerged as integrative prognostic tools for solid tumors. These models are based on the theoretical premise that cancer progression results from the coordinated interaction between immune activation, nutritional depletion, and metabolic stress within the tumor–host axis rather than from a single inflammatory pathway. Numerous studies in glioma have demonstrated that elevated SII, SIRI, PIV, and GINI values or decreased HALP scores are significantly associated with shorter progression-free survival (PFS) and OS [23,24,25,26,27]. Collectively, these findings have reshaped the prognostic landscape of neuro-oncology by reinforcing the concept that systemic immune–metabolic disequilibrium mirrors molecular aggressiveness and adverse clinical behavior. This evolving evidence provides a strong conceptual and methodological foundation for developing next-generation composite biomarkers that integrate cellular turnover, functional reserves, and oxidative stress to refine survival prediction in grade 4 adult-type diffuse gliomas.

Building upon growing evidence that systemic biomarkers can reflect both tumor biology and host response, we developed the HALLMOUNT score, a novel and comprehensive prognostic model for patients with high-grade gliomas. This integrative index encompasses nine systemic biomarkers and functional parameters to capture the multidimensional interaction between the tumor and host, incorporating immune activity, nutritional status, systemic inflammation, oxidative stress, and overall functional capacity. Each component represents a distinct yet complementary biological domain: H for hemoglobin (anemia and oxygen-carrying capacity), A for albumin (nutritional reserve and inflammation), L for lactate dehydrogenase (cellular turnover and tumor burden), L for lymphocyte count (immune competence), M for monocyte count (immunosuppressive activity), O for overall condition measured by the ECOG PS (functional status), U for uric acid (oxidative stress), N for neutrophil count (systemic inflammatory load), and T for thrombocyte count (tumor-associated inflammation and vascular reactivity). By integrating these parameters into a single quantitative framework, the HALLMOUNT score provides a holistic and biologically grounded assessment of patient prognosis. The present study was designed to evaluate the prognostic and predictive significance of the HALLMOUNT score in a large cohort of patients with grade 4 adult-type diffuse glioma and to determine its comparative performance against established systemic inflammation-based indices in predicting survival outcomes.

## 2. Materials and Methods

### 2.1. Study Design, Participant Selection, Data Collection, and Clinical Follow-Up

In this retrospective study, clinical data from 309 patients treated and monitored at the Oncology Department of the University of Health Sciences Antalya Education and Research Hospital (UHSAERH) between June 2015 and February 2025 were analyzed. All patients had a pathologically confirmed diagnosis of grade 4 adult-type diffuse glioma based on the criteria of the fifth edition of the WHO Classification of Tumors of the CNS, published in 2021 [1]. Standard treatment was administered in accordance with the Stupp protocol [6], comprising maximal safe surgical resection followed by concurrent radiotherapy (RT) with temozolomide (TMZ) and subsequent adjuvant TMZ. Preoperative evaluations, including complete peripheral blood counts and comprehensive biochemical analyses, were conducted one week before surgery.

**Figure 1:**
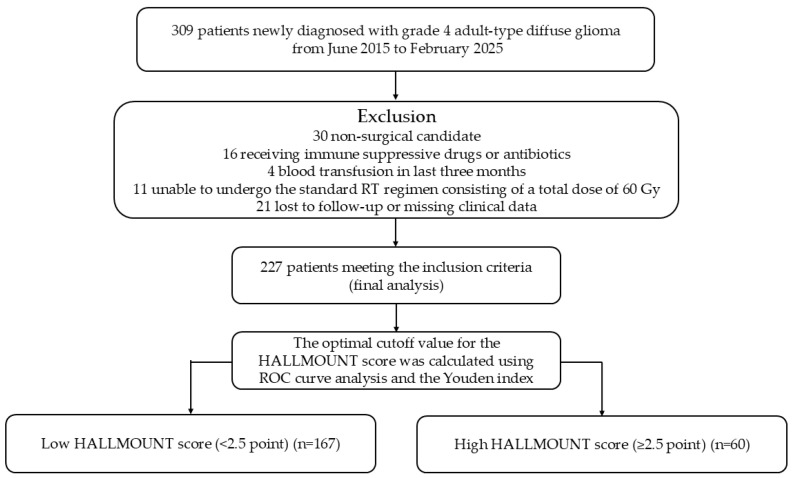
Cohort flow diagram of the study population.

Thirty patients who did not undergo surgical resection, 11 patients who were unable to receive the standard focal RT regimen of 60 Gy, and 21 patients with incomplete clinical follow-up data were excluded from the study. In addition, 9 patients receiving steroid therapy for chronic inflammatory diseases, 7 patients using antibiotics for acute infections, and 4 patients with a history of blood transfusion within the preceding 3 months were excluded, as these conditions could cause transient fluctuations in immune-inflammatory biomarkers and potentially lead to misinterpretation. Consequently, the final cohort comprised 227 patients with complete clinicopathological and laboratory data (Figure 1).

Data on age, sex, body mass index (BMI), comorbidities, smoking status, Eastern Cooperative Oncology Group (ECOG) PS, preoperative brain magnetic resonance imaging (MRI) tumor size, tumor lateralization, tumor focality, tumor-involved lobe, surgical procedure (gross total resection [GTR] vs. non-GTR), IDH mutation status, *alpha thalassemia/mental retardation syndrome X-linked* (*ATRX*) loss, *O6-methylguanine-DNA methyltransferase* (*MGMT*) promoter methylation status, response to adjuvant therapy, progression status, treatments administered at progression, and survival outcomes.

Following the completion of radiotherapy, interim evaluations for patients scheduled to receive adjuvant TMZ were performed in accordance with the National Comprehensive Cancer Network (NCCN) guidelines, using biochemical tests and serial MRI scans. During clinical follow-up, radiological responses were categorized as complete response (CR), partial response (PR), minor response (MR), stable disease (SD), or progressive disease (PD), according to the Response Assessment in Neuro-Oncology (RANO 2.0) criteria for high-grade gliomas revised in 2023 [28]. PFS was defined as the interval from the date of pathological diagnosis to progression, death, or the last follow-up, whichever occurred first. OS was defined as the interval from the date of pathological diagnosis to death from any cause or last follow-up.

### 2.2. Definition of the HALLMOUNT Model

In this study, we introduced and evaluated a novel prognostic scoring system, the HALLMOUNT score (hemoglobin–albumin–lactate dehydrogenase–lymphocyte–monocyte–overall condition–uric acid–neutrophil–thrombocyte), designed to integrate hematologic, biochemical, and clinical performance parameters into a single composite measure reflecting systemic inflammation, nutritional status, immune competence, tumor burden, and overall patient condition. The index comprises nine components: hemoglobin (<12 g/dL), albumin (<3.5 g/dL), lactate dehydrogenase (LDH ≥ 220 U/L), lymphocyte count (<1000/μL), monocyte count (≥600/μL), overall functional status assessed by the Eastern Cooperative Oncology Group (ECOG) performance status (≥2), uric acid (>7 mg/dL for males, >6 mg/dL for females), neutrophil count (≥6000/μL), and thrombocyte count (≥400,000/μL, or literature-based cut-off). Each parameter meeting the predefined threshold was assigned 1 point, yielding a total score ranging from 0 to 9, with higher scores indicating a poorer prognostic profile. To ensure consistency and enable prognostic comparisons, established immune–inflammatory and nutrition-based indices were calculated from pretreatment laboratory values as follows: NLR = neutrophils/lymphocytes [29], PIV = (NLR × monocytes × platelets) [30], and CAR = C-reactive protein/albumin [31].

The selection of thresholds for each HALLMOUNT parameter followed a standardized three-step approach: (a) established evidence-based cutoffs from prior studies on inflammation-nutrition indices and glioma prognostics (e.g., LDH ≥ 220 U/L, albumin <3.5 g/dL, lymphocyte < 1000/µL), (b) laboratory-defined institutional reference ranges for hematologic and biochemical variables to ensure clinical applicability, and (c) ROC–Youden index optimization within our cohort. For parameters where the literature and institutional standards were aligned, the existing thresholds were retained; for others, the data-driven Youden cutoffs were applied.

Equal weighting (1 point per parameter) was intentionally selected to create a simple, clinically practical index. To evaluate whether disproportionate weighting was necessary, we performed an exploratory multivariable Cox regression and compared standardized β-coefficients of all nine variables. No single parameter demonstrated a sufficiently dominant coefficient to justify differential weighting. Additionally, to assess multicollinearity among HALLMOUNT and other inflammatory indices (NLR, CAR, PIV), variance inflation factors (VIFs) were calculated for all variables incorporated into the multivariable Cox models. All VIF values were below 5, indicating no significant multicollinearity. Prior multidimensional indices used in solid tumors (PIV, SII, SIRI, HALP, GINI) similarly rely on equal-weight categorical components rather than continuous regression-based scoring to maximize bedside usability.

### 2.3. HALLMOUNT Score Calculation Algorithm

Each of the nine components was coded as a binary variable (0 or 1 point) based on predefined thresholds grounded in the literature, institutional reference ranges, or ROC–Youden optimization. The final score equals the sum of all positive parameters, ranging from 0 to 9, with higher scores indicating worse systemic inflammatory, nutritional, metabolic, and functional status (Table 1).

To enhance the practical interpretability of the HALLMOUNT model, two representative clinical scenarios demonstrating the scoring process are provided below.

Example 1 (low-risk profile; HALLMOUNT = 2): A 54-year-old male with grade 4 adult-type diffuse glioma presents with preserved functional status (ECOG PS 1), normal hemoglobin (13.1 g/dL) and albumin (4.0 g/dL) levels, and an LDH value within the institutional reference range (190 U/L). His absolute lymphocyte count is 1150/µL and monocyte count is 720/µL, both below thresholds associated with immunosuppression or myeloid activation. Neutrophil and platelet counts are similarly unremarkable (5400/µL and 310,000/µL, respectively). Two parameters exceed predefined cut-off values: a mildly elevated uric acid level at 7.4 mg/dL (scoring 1 point for male sex) and a monocyte count above 600/µL (scoring 1 additional point). All remaining components receive a score of 0, resulting in a total HALLMOUNT score of 2. This configuration reflects a largely favorable systemic profile with minimal inflammatory or metabolic disturbance.

Example 2 (high-risk profile; HALLMOUNT = 5): A 68-year-old female patient demonstrates substantial systemic vulnerability. She presents with anemia (hemoglobin 10.4 g/dL), hypoalbuminemia (3.1 g/dL), and elevated LDH (295 U/L), each contributing 1 point. Her lymphocyte count is markedly reduced (850/µL), consistent with impaired adaptive immunity, and her ECOG PS is 2, indicating diminished functional reserve; both fulfill scoring criteria. In contrast, neutrophil and platelet counts fall below their respective thresholds, and the serum uric acid level is within the normal female range (5.8 mg/dL), each receiving a score of 0. The cumulative HALLMOUNT score is 5, representing a high-risk inflammatory, nutritional, and metabolic profile aligned with her clinically fragile condition.

These examples demonstrate how the HALLMOUNT framework synthesizes multiple biologically relevant parameters into a cohesive prognostic model that can be applied across diverse clinical presentations.

### 2.4. Ethics Statement

The study protocol was formally reviewed and approved by the Institutional Review Board of UHSAERH (Approval No. 2025-339) on 11 September 2025, in accordance with the Declaration of Helsinki (1964, revised 2024). Given the retrospective nature of the study, the board waived the need for informed consent and all patient data were de-identified to maintain confidentiality.

### 2.5. Statistical Analysis

All statistical analyses were performed using the SPSS software (version 27.0; IBM Corp., Armonk, NY, USA). Data distribution was assessed using the Kolmogorov–Smirnov test. Normally distributed variables are expressed as mean ± standard deviation, and non-normally distributed variables are expressed as median (range). Receiver operating characteristic (ROC) curves were constructed to evaluate the prognostic performance of HALLMOUNT, NLR, CAR, and PIV. Area under the curve (AUC) values and 95% confidence intervals (CIs) were obtained using SPSS syntax procedures. To formally compare the discriminatory capacity of these indices, pairwise AUC comparisons were conducted using the nonparametric DeLong test, implemented in MedCalc Statistical Software, version 20.0 (MedCalc Software Ltd., Ostend, Belgium). Z-statistics and two-sided *p*-values were calculated for each comparison. Time-dependent ROC analysis was conducted using a validated landmark-based approach. For each inflammatory index, event status was redefined at 12- and 24-month landmarks by coding patients who had died by the given time as events and those alive beyond the time point as non-events. ROC curves at each landmark were generated in SPSS to obtain AUC values, 95% CIs, optimal cut-off points (Youden index), sensitivity, and specificity. To assess global, time-integrated discriminative ability, a Harrell-type C-index was calculated using a user-defined SPSS macro that compares all permissible patient pairs and generates bootstrap-based 95% CIs. This combined framework enabled consistent evaluation of short-term, intermediate-term, and overall prognostic discrimination across all indices. Propensity score methods, including inverse probability of treatment weighting (IPTW), were not performed, as the study aimed to evaluate prognostic rather than comparative effects. Treatment allocation (radiotherapy versus temozolomide) inherently reflected established clinical decision-making based on age, comorbidity, MGMT status, ECOG performance status, and tumor burden. Because this indication-driven pattern violates key assumptions of covariate overlap and positivity, propensity-based weighting would yield unstable or uninterpretable estimates and was deemed methodologically inappropriate for the prognostic focus of the study. Because this study did not involve the development or fitting of a multivariable prediction model, bootstrap-based optimism correction was not applied. The discrimination metrics used including landmark AUC values and Harrell’s C-index were derived directly from observed survival outcomes and are therefore not susceptible to coefficient overfitting. Accordingly, the application of bootstrap optimism correction was deemed methodologically inappropriate given the aims and analytic structure of this prognostic stratification study. Associations between HALLMOUNT and clinical variables were examined using the chi-square or Fisher’s exact tests. OS and PFS were estimated using the Kaplan–Meier method and compared using the log-rank test. Survival predictors were first explored using univariate Cox regression, and variables that achieved statistical significance were subsequently entered into multivariate Cox models. Statistical significance was set at a two-tailed *p*-value < 0.05.

### 2.6. Statistical Hypothesis

In accordance with recommendations for transparent reporting of prognostic studies, we explicitly defined the statistical hypotheses prior to analysis.

Null hypothesis (H0):

(1) The HALLMOUNT score has no prognostic association with overall survival OS or PFS in patients with grade 4 adult-type diffuse glioma.
(2) The prognostic performance of the HALLMOUNT score does not differ from established inflammatory indices, including the NLR, CAR, and PIV.

Alternative hypothesis (H1):

(1) The HALLMOUNT score is associated with OS and PFS.
(2) Its prognostic performance differs from that of NLR, CAR, and PIV.

A “difference” rather than “superiority” framework was chosen for comparative analyses because the study was not designed to formally test superiority, and the observed discriminative performance of CAR was slightly higher than that of HALLMOUNT. All analyses were conducted in accordance with this predefined hypothesis structure.

## 3. Results

In total, 227 patients were included in this study. The median age was 60 years (range: 21–88 years), and 49.3% of the patients were younger than 60 years. The cohort included 129 males (56.8%) and 98 females (43.2%). Comorbidities were present in 91 patients (40.1%) and 76 (33.5%) were current smokers. An ECOG PS ≥ 2 was observed in 47 patients (20.7%). Tumors were most commonly located in the parietal lobe and least frequently in the cerebellum, whereas multifocal disease was present in 50 patients (22.0%). A tumor size ≥ 50 mm was recorded in 80 patients (35.2%). IDH mutations were identified in 47 cases (20.7%), *ATRX* loss in 77 cases (33.9%), and *MGMT* positivity in 49 cases (21.6%). GTR was achieved in 119 (52.4%) patients. Adjuvant radiotherapy and chemotherapy were administered to 90.7% and 82.4% of the patients, respectively, with a complete response rate of 32.1%. The clinical, demographic, and molecular features of the cohort are presented in Table 2, along with comparisons between patients classified into the low and high HALLMOUNT score groups.

### 3.1. ROC Analysis and Cut-Off Classification

**Figure 2:**
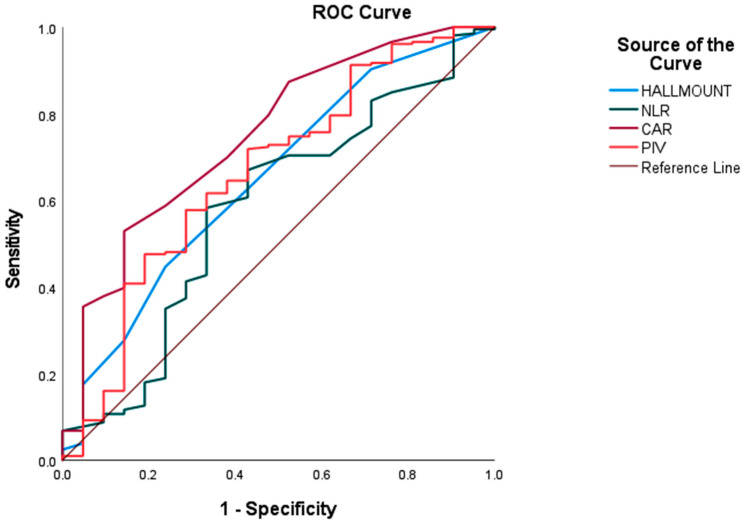
ROC-based comparison of HALLMOUNT and conventional immune–nutritional indices for mortality prediction in grade 4 adult-type diffuse gliomas.

ROC curve analysis demonstrated a significant predictive performance for HALLMOUNT (AUC = 0.650, 95% CI: 0.525–0.775; *p* = 0.024), NLR (AUC = 0.653, 95% CI: 0.522–0.785; *p* = 0.021), CAR (AUC = 0.750, 95% CI: 0.639–0.861; *p* < 0.001), and PIV (AUC = 0.664, 95% CI: 0.534–0.795; *p* = 0.013), with CAR yielding the highest AUC value. Sensitivity and specificity were 44.7–76.2% for HALLMOUNT, 69.4–66.7% for NLR, 52.9–85.7% for CAR, and 57.8–71.4% for PIV. Based on the Youden index-derived cut-offs, 150 patients (66.1%) had NLR ≥ 2.25, 112 (49.3%) had CAR ≥ 0.65, 126 (55.5%) had PIV ≥ 496.9, and 60 (26.4%) had HALLMOUNT ≥ 2.5 (Figure 2, Table 3).

Pairwise AUC comparisons using the DeLong test revealed that HALLMOUNT demonstrated significantly superior discriminatory performance compared with NLR at both 12 months (Z = 4.21, *p* < 0.001) and 24 months (Z = 3.38, *p* < 0.001). In contrast, the differences between HALLMOUNT and CAR (12-month *p* = 0.53; 24-month *p* = 0.30) and between HALLMOUNT and PIV (12-month *p* = 0.85; 24-month *p* = 0.65) were not statistically significant, indicating comparable prognostic accuracy among these indices. CAR and PIV also showed significantly better performance than NLR at both time points. These findings position HALLMOUNT, CAR, and PIV as the strongest prognostic markers, whereas NLR exhibited the weakest discriminative ability.

Landmark-based time-dependent ROC analysis demonstrated that HALLMOUNT consistently exhibited the strongest discriminatory performance. The 12-month AUC was 0.86 (95% CI, 0.82–0.91), indicating excellent early mortality prediction, and prognostic accuracy remained robust at 24 months (AUC = 0.80; 95% CI, 0.73–0.87). CAR and PIV showed similar high-level performance, whereas NLR demonstrated limited predictive value at both time points. The global Harrell C-index further confirmed the stability of HALLMOUNT as a long-term prognostic classifier (C-index = 0.78; 95% CI, 0.75–0.81), outperforming NLR and comparable to CAR and PIV. These findings highlight the superior and consistent prognostic capacity of HALLMOUNT across both short-term and overall survival endpoints (Table 4).

### 3.2. Associations of HALLMOUNT with Clinical, Molecular, and Treatment Response Parameters

A high HALLMOUNT score (≥2.5) was significantly associated with older age (≥60 years: 70.0% vs. 43.7%, *p* < 0.001), presence of comorbidities (55.0% vs. 34.7%, *p* = 0.005), and poorer performance status (ECOG ≥ 2:50.0% vs. 10.2%, *p* < 0.001), whereas sex distribution did not differ between groups. IDH mutations were markedly more frequent in patients with low HALLMOUNT scores (25.7% vs. 6.7%, *p* < 0.001), whereas ATRX loss was not significantly associated. The likelihood of achieving GTR was higher among low HALLMOUNT patients (57.5% vs. 38.3%, *p* = 0.021). Similarly, adjuvant radiotherapy and chemotherapy were delivered significantly less often in the high HALLMOUNT group (both *p* < 0.001). patients with high HALLMOUNT scores exhibited a substantially greater incidence of progressive disease (68.3% vs. 27.0%) and fewer complete or partial responses (20.0% vs. 36.5%; *p* < 0.001). A high HALLMOUNT status was also closely correlated with elevated CAR and PIV levels (both *p* < 0.001), indicating a consistent association between systemic inflammation and impaired clinical outcomes.

### 3.3. Survival Analyses

**Figure 3:**
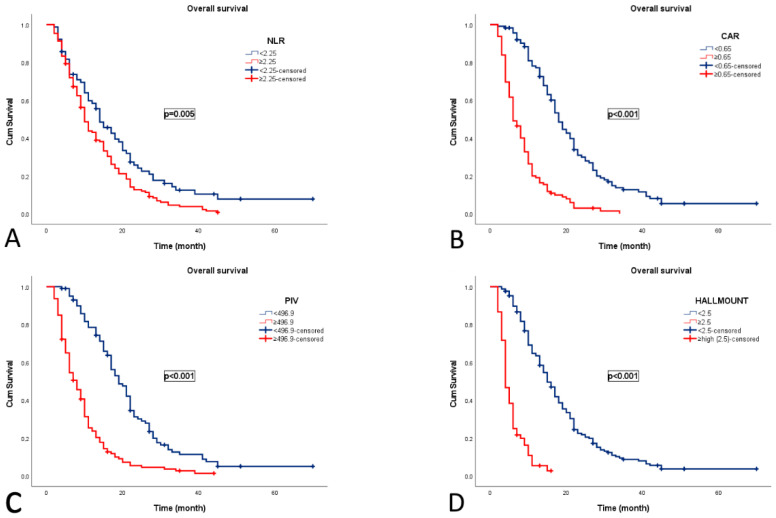
Kaplan–Meier curves for overall survival according to composite indices in patients with grade 4 adult-type diffuse gliomas. (**A**) NLR, neutrophil-to-lymphocyte ratio; (**B**) CAR, C-reactive protein-to-albumin ratio; (**C**) PIV, pan-immune–inflammation value; (**D**) HALLMOUNT score.

During a median follow-up of 16.8 months, 206 patients (90.7%) died, with a median OS of 11 months (95% CI: 9.4–12.6). Median OS was significantly reduced in patients with NLR ≥ 2.25 (10 vs. 14 months, *p* = 0.005), CAR ≥ 0.65 (6 vs. 18 months, *p* < 0.001), PIV ≥ 496.9 (8 vs. 19 months, *p* < 0.001), and HALLMOUNT ≥ 2.5 (4 vs. 15 months, *p* < 0.001) (Figure 3).

**Figure 4:**
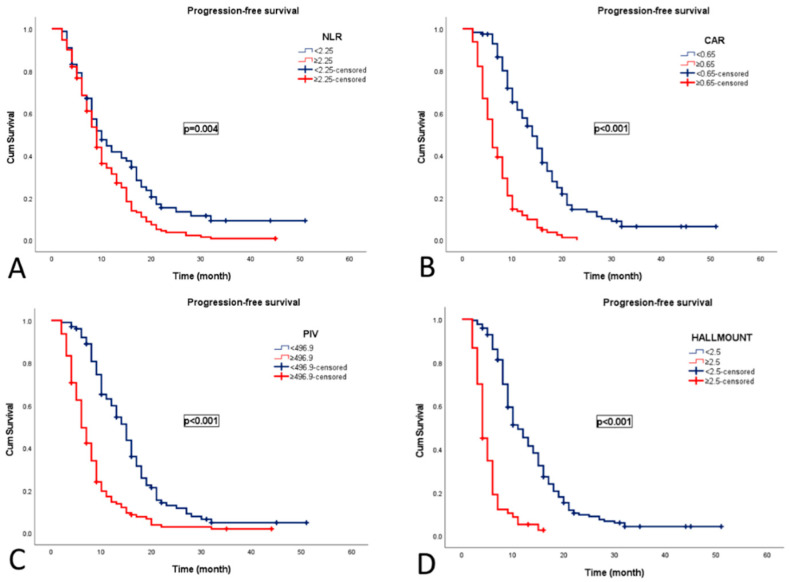
Kaplan–Meier curves for progression-free survival according to composite indices in patients with grade 4 adult-type diffuse gliomas. (**A**) NLR, neutrophil-to-lymphocyte ratio; (**B**) CAR, C-reactive protein-to-albumin ratio; (**C**) PIV, pan-immune–inflammation value; (**D**) HALLMOUNT score.

Progression occurred in 212 patients (93.4%) with a median PFS of 9 months (95% CI: 8.8–9.1). Median PFS was significantly shorter in patients with NLR ≥ 2.25 (9 vs. 10 months, *p* = 0.004), CAR ≥ 0.65 (6 vs. 14 months, *p* < 0.001), PIV ≥ 496.9 (6 vs. 15 months, *p* < 0.001), and HALLMOUNT ≥ 2.5 (4 vs. 11 months, *p* < 0.001) (Figure 4).

### 3.4. Cox Regression Analyses

In univariate Cox regression analysis, adverse prognostic factors for OS included advanced age (HR = 1.542, *p* = 0.002), poor performance status (ECOG ≥ 2; HR = 2.440, *p* < 0.001), multifocality (HR = 1.770, *p* = 0.001), and IDH wild-type status (HR = 3.426, *p* < 0.001). The protective variables included *ATRX* loss (HR = 0.672, *p* = 0.008), *MGMT* promoter methylation (HR = 0.764, *p* = 0.003), GTR (HR = 0.553, *p* < 0.001), adjuvant radiotherapy (HR = 0.119, *p* < 0.001), and adjuvant chemotherapy (HR = 0.137, *p* < 0.001). Elevated NLR, CAR, PIV, and HALLMOUNT were significantly associated with increased mortality risk (HR = 1.504, 3.645, 3.020, and 6.361, respectively; all *p* < 0.01) (Table 5).

Similar patterns were observed for PFS. Adverse factors included older age (HR = 1.391, *p* = 0.019), ECOG ≥ 2 (HR = 2.315, *p* < 0.001), multifocality (HR = 1.638, *p* = 0.003), and IDH wild-type status (HR = 2.903, *p* < 0.001), whereas *ATRX* loss, *MGMT* methylation, GTR, and adjuvant therapy were protective (all *p* < 0.01). A higher NLR (HR = 1.518, *p* = 0.007), CAR (HR = 3.507, *p* < 0.001), PIV (HR = 2.547, *p* < 0.001), and HALLMOUNT (HR = 4.894, *p* < 0.001) were significantly associated with shorter PFS (Table 5).

In multivariate analysis, independent predictors of poor OS were multifocality (hazard ratio [HR] = 1.626, *p* = 0.006) and IDH wild-type status (HR = 2.379, *p* < 0.001), whereas GTR (HR = 0.631, *p* = 0.003), adjuvant radiotherapy (HR = 0.211, *p* < 0.001), and adjuvant chemotherapy (HR = 0.514, *p* = 0.010) retained independent protective significance. Elevated NLR (hazard ratio [HR] = 1.440, *p* = 0.028), CAR (HR = 2.467, *p* < 0.001), PIV (HR = 1.996, *p* < 0.001), and HALLMOUNT (HR = 2.776, *p* < 0.001) remained independently associated with inferior OS (Table 6).

For PFS, IDH wild-type tumors (hazard ratio [HR] = 1.917, *p* = 0.006) independently predicted shorter survival, whereas GTR (HR = 0.596, *p* = 0.001), adjuvant radiotherapy (HR = 0.260, *p* < 0.001), and chemotherapy (HR = 0.580, *p* = 0.033) were independently protective. Treatment response was also associated with progression risk (HR = 1.252, *p* = 0.022). Among the hematological indices, CAR (HR = 2.439, *p* < 0.001), PIV (HR = 1.800, *p* = 0.001), and HALLMOUNT (HR = 2.763, *p* < 0.001) retained independent prognostic significance for PFS, whereas NLR was not statistically significant (HR = 1.280, *p* = 0.135) (Table 6).

## 4. Discussion

Grade 4 adult-type diffuse gliomas are the most prevalent primary malignant tumors of the central nervous system and are characterized by extensive heterogeneity, invasive growth, and resistance to standard therapy [2,5]. Despite advancements in molecular classification and multimodal treatment [32,33], the prognosis remains dismal [6,8]. Tumor progression is strongly influenced by the interplay between glioma cells and the host immune–inflammatory milieu, in which chronic inflammation, metabolic stress, and impaired nutritional status jointly shape therapeutic resistance and disease trajectory [13].

This study introduces the HALLMOUNT score, a novel multidimensional prognostic index integrating hematological, biochemical, and clinical variables, to reflect the interplay between tumor biology and the host’s systemic response in grade 4 adult-type diffuse gliomas. Elevated HALLMOUNT scores were significantly associated with adverse clinical features, including advanced age, comorbidities, poor ECOG PS, and IDH wild-type phenotype. Although its sensitivity for mortality prediction was only moderate and its overall performance was inferior to that of CAR (0.750 vs. 0.650), its high specificity nonetheless supports its utility in accurately identifying high-risk patient subgroups. Patients with high scores showed markedly poorer treatment responses and survival outcomes, indicating that the index effectively captures systemic inflammation, metabolic imbalance, and tumor aggressiveness. Moreover, its role as an independent negative prognostic factor for both OS and PFS, comparable to that of CAR and PIV, underscores its robustness. The strong correlations with established inflammatory and nutritional indices further validated its comprehensive prognostic relevance.

Several studies have evaluated inflammation- and nutrition-based indices as prognostic markers for high-grade central nervous system tumors. Evolving from simple ratios such as NLR [19,21] and CAR [22] to composite indices such as PIV [25], GINI [26], and HALP [27], these studies consistently associated heightened systemic inflammation and nutritional imbalance with poor survival. In line with this evidence, our results confirmed the prognostic relevance of NLR, CAR, and PIV in glioma outcomes. The HALLMOUNT score demonstrated strong predictive performance for both OS and PFS and retained independent prognostic significance in the multivariate analyses. Notably, the inverse association between the HALLMOUNT score and protective molecular features, including IDH mutations and *MGMT* promoter methylation, suggests that the score captures both systemic vulnerability and intrinsic tumor aggressiveness.

The components of the HALLMOUNT score reflect the critical biological and systemic mechanisms that contribute to glioma progression. Elevated LDH denotes increased glycolytic flux and tumor metabolic activity in accordance with the “Warburg effect,” while simultaneously indicating hypoxia-driven angiogenesis and an enhanced tumor burden [34]. Hypoalbuminemia and anemia serve as indicators of chronic systemic inflammation and impaired oxygen transport capacity, conditions that aggravate hypoxic stress and reduce treatment tolerance [18,35]. Lymphopenia reflects diminished cytotoxic and helper T-cell function, signifying weakened antitumor immune surveillance, whereas monocytosis and neutrophilia promote tumor invasion and immune escape through the activation of TAMs and formation of neutrophil extracellular traps [12,13]. Thrombocytosis facilitates endothelial remodeling and neovascularization via platelet-derived growth factors, such as PDGF and VEGF, thereby promoting tumor vascularization [17]. Furthermore, elevated uric acid levels, representing oxidative stress and disturbed purine metabolism, are linked to DNA damage, the activation of proinflammatory pathways, and microglial stimulation within the tumor microenvironment [36]. Lastly, poor ECOG performance status reflects systemic frailty and neurological impairment, highlighting the significance of the host functional reserve in determining disease trajectory [37]. Collectively, the HALLMOUNT score integrates tumor-intrinsic aggressiveness with host systemic tolerance, providing a comprehensive and clinically relevant biomarker for glioma biology and patient outcomes.

Clinically, the HALLMOUNT score may provide a simple and cost-efficient means of patient stratification before surgical and adjuvant therapies. Although high HALLMOUNT scores were strongly associated with reduced likelihood of receiving radiotherapy and temozolomide, this treatment imbalance should primarily be interpreted as a reflection of systemic frailty and impaired treatment tolerance rather than as a classical confounder. Patients with elevated inflammatory and nutritional derangements frequently fail to meet the clinical criteria for intensive multimodal therapy. Accordingly, reduced RT/TMZ administration likely acts as a mediating pathway linking physiologic vulnerability to inferior survival. While propensity-score weighting or matching techniques could theoretically adjust for such imbalances, the modest sample size, high event rate, retrospective design, and incomplete molecular annotation may limit their stability and interpretability. Therefore, treatment variables were incorporated into the multivariable Cox models rather than subjected to causal weighting. High-risk patients identified by elevated HALLMOUNT scores despite standard multimodal therapy may warrant early enrollment in clinical trials or supportive strategies to enhance systemic resilience. In contrast, those with low scores may be suitable for more intensive curative-intent approaches. Incorporating the HALLMOUNT score into a clinical trial design could enhance prognostic uniformity among participants, thereby improving the accuracy of efficacy assessments of emerging immunotherapeutic or metabolic interventions.

The correlation between high HALLMOUNT scores and poor outcomes supports the concept that glioma progression arises from intertwined immune–metabolic dysregulation. Chronic inflammation drives myeloid reprogramming, whereas metabolic stress enhances immunosuppressive signaling via lactate and adenosine accumulation. Uric acid–induced NOD-, LRR-, and pyrin domain-containing protein 3 (NLRP3) activation and neutrophil infiltration further reinforced this tumor-promoting milieu [38]. By incorporating LDH and uric acid, the HALLMOUNT model bridges systemic metabolic stress with local immune dysfunction, capturing their combined influence on tumor biology. These findings align with the molecular evidence that metabolic reprogramming and immune exhaustion synergistically sustain glioma aggressiveness and resistance to therapy.

Future studies should prospectively validate the HALLMOUNT score in multicenter, molecularly defined cohorts to address the limitations of this single-center, retrospective analysis, and potential laboratory variability. Automated analyzers calibrated to international standards minimize inter-assay variability. Although formal inter-laboratory coefficient of variation data were not available, analytical variability is expected to remain within acceptable ranges for all measured biomarkers. A substantial proportion of *MGMT* promoter methylation data (48.9%) was missing, largely due to the limited availability of routine molecular testing during the earlier years of the study period. Because the mechanism of missingness was driven by institutional and temporal factors rather than occurring completely at random, multiple imputation was considered inappropriate; imputing biologically non-random molecular variables could introduce additional bias. Integration with radiomic, metabolomic, and genomic markers, such as *MGMT*, *TERT*, and *H3F3A G34R/V* may enable composite models with enhanced prognostic precision. This study did not incorporate internal validation procedures such as bootstrapping or k-fold cross-validation. Given the retrospective design, high event rate, and incomplete molecular annotation, internal resampling techniques may have produced unstable estimates and limited clinical interpretability. Instead, VIF and multivariable Cox regression were utilized to minimize overfitting within the development cohort. Nonetheless, we acknowledge that the absence of internal validation represents a methodological limitation. Future multicenter prospective studies with balanced molecular profiling are essential to externally validate and refine the HALLMOUNT score. Longitudinal monitoring of HALLMOUNT components could allow real-time assessment of systemic dynamics, support adaptive therapy, and early risk identification. Despite incomplete molecular data and variability in advanced-line treatments, the multidimensional HALLMOUNT model provides a biologically grounded framework linking tumor biology with host systemic status, underscoring its potential as a practical tool for risk stratification and personalized therapy in neurooncology.

## 5. Conclusions

The HALLMOUNT score provides a clinically actionable multidimensional biomarker that captures both tumor aggressiveness and host systemic vulnerability in grade 4 adult-type diffuse gliomas. High scores identify patients at greatest risk for poor survival and treatment intolerance, support tailored therapeutic strategies, and early trial enrollment. This score offers a biologically grounded framework for precision neuro-oncology by integrating immune, metabolic, and functional parameters. Prospective validation and incorporation of molecular and radiomic markers could further enhance its utility, enabling dynamic risk stratification and more personalized adaptive therapies.

## Figures and Tables

**Table 1:** Scoring algorithm of the HALLMOUNT model, including component thresholds and point assignments for the nine hematologic, biochemical, and functional parameters.

Component	Threshold	Score
Hemoglobin	<12 g/dL	1
Albumin	<3.5 g/dL	1
LDH	≥220 U/L	1
Lymphocytes	<1000/µL	1
Monocytes	≥600/µL	1
ECOG PS	≥2	1
Uric acid	>7 mg/dL (male), >6 mg/dL (female)	1
Neutrophils	≥6000/µL	1
Thrombocytes	≥400,000/µL	1

**Table 2:** Clinicopathological and demographic characteristics of the patients, stratified by low and high HALLMOUNT score groups (*n = 227*).

Variables, *n (%)*			HALLMOUNT Score		*p*
			Low (<2.5) *, n (%)	High (≥2.5) *, n (%)	
Age	<60	112 (49.3)	94 (56.3)	18 (30.0)	<0.001
	≥60	115 (50.7)	73 (43.7)	42 (70.0)	
Sex	Male	129 (56.8)	97 (58.1)	32 (53.3)	0.313
	Female	98 (43.2)	70 (41.9)	28 (46.7)	
Comorbidity	No	136 (59.9)	109 (65.3)	27 (45.0)	0.005
	Yes	91 (40.1)	58 (34.7)	33 (55.0)	
Smoking	No	151 (66.5)	108 (64.7)	43 (71.7)	0.205
	Yes	76 (33.5)	59 (35.3)	17 (28.3)	
ECOG PS	0–1	180 (79.3)	150 (89.8)	30 (50.0)	<0.001
	≥2	47 (20.7)	17 (10.2)	30 (50.0)	
Lateralization	Left	119 (52.4)	85 (50.9)	34 (56.7)	0.269
	Right	108 (47.6)	82 (49.1)	26 (43.3)	
Tumor localization	Frontal	63 (27.8)	43 (25.7)	20 (33.3)	0.720
	Occipital	23 (10.1)	18 (10.8)	5 (8.3)	
	Parietal	85 (37.4)	62 (37.1)	23 (38.3)	
	Cerebellar	2 (0.9)	1 (0.6)	1 (1.7)	
	Temporal	54 (23.8)	43 (25.7)	11 (18.3)	
Tumor focality	Unifocal	177 (78.0)	132 (79.0)	45 (75.0)	0.316
	Multifocal	50 (22.0)	35 (21.0)	15 (25.0)	
Tumor size	<50 mm	147 (64.8)	110 (65.9)	37 (61.7)	0.333
	≥50 mm	80 (35.2)	57 (34.1)	23 (38.3)	
IDH mutation	Mutant	47 (20.7)	43 (25.7)	4 (6.7)	<0.001
	Wild	180 (79.3)	124 (74.3)	56 (93.3)	
ATRX loss	No	150 (66.1)	106 (63.5)	44 (73.3)	0.109
	Yes	77 (33.9)	61 (36.5)	16 (26.7)	
MGMT promoter methylation	Negative	67 (29.5)	40 (24.0)	27 (45.0)	0.134
	Positive	49 (21.6)	46 (27.5)	3 (5.0)	
	Unknown	111 (48.9)	81 (48.5)	30 (50.0)	
Type of surgery	Non-GTR	108 (47.6)	71 (42.5)	37 (61.7)	0.021
	GTR	119 (52.4)	96 (57.5)	23 (38.3)	
Adjuvant radiotherapy	No	21 (9.3)	5 (3.0)	16 (26.7)	<0.001
	Yes	206 (90.7)	162 (97.0)	44 (73.3)	
Adjuvant chemotherapy	No	40 (17.6)	6 (3.6)	34 (56.7)	<0.001
	Yes	187 (82.4)	161 (96.4)	26 (43.3)	
Treatment response ^#^	CR	73 (32.1)	61 (36.5)	12 (20.0)	<0.001
	PR + MR + SD	68 (30.0)	61 (36.5)	7 (11.7)	
	PD	86 (37.9)	45 (27.0)	41 (68.3)	
NLR, (2.25) *	Low	77 (33.9)	59 (35.3)	18 (30.0)	0.280
	High	150 (66.1)	108 (64.7)	42 (70.0)	
CAR, (0.65) *	Low	115 (50.7)	102 (61.1)	13 (21.7)	<0.001
	High	112 (49.3)	65 (38.9)	47 (78.3)	
PIV, (496.9) *	Low	101 (44.5)	98 (58.7)	3 (5.0)	<0.001
	High	126 (55.5)	69 (41.3)	57 (95.0)	

Abbreviations: ECOG PS, Eastern Cooperative Oncology Group performance status; IDH, Isocitrate dehydrogenase; ATRX, Alpha thalassemia/mental retardation syndrome X-linked; MGMT, O6-methylguanine-DNA methyltransferase; Non-GTR, cases where only partial resection was achieved; cases involving biopsy alone were excluded; GTR, Gross total resection; # (based on the findings from the initial response assessment conducted at week 12 following radiotherapy according to RANO 2.0) [28]; CR, complete response; PR, partial response; MR, minor response; SD, stable disease; PD, progressive disease; NLR, neutrophil-to-lymphocyte ratio; CAR, C-reactive protein-to-albumin ratio; PIV, Pan-immune–inflammation value; *, cutoff values determined for each index.

**Table 3:** Comparison of AUC values for each index by ROC curve analysis.

	AUC	Std. Error	95% CI (Lower-Upper)	*p* Value	Sensitivity	Specificity	Cut Off
HALLMOUNT	0.650	0.064	0.525–0.775	0.024	44.7%	76.2%	2.50
NLR	0.653	0.067	0.522–0.785	0.021	69.4%	67%	2.25
CAR	0.750	0.057	0.639–0.861	<0.001	52.9%	86%	0.650
PIV	0.664	0.067	0.534–0.795	0.013	57.8%	71%	496.9

Abbreviations: AUC, area under the curve; CI, confidence interval; Std, standard deviation; NLR, neutrophil-to-lymphocyte ratio; CAR, C-reactive protein-to-albumin ratio; PIV, pan-immune–inflammation value.

**Table 4:** Time-dependent ROC metrics and global C-Index demonstrating the prognostic performance of HALLMOUNT versus established inflammation-based indices.

Marker	12-mo AUC (95% CI)	Cut-Off	Sensitivity	Specificity	*p*-Value	24-mo AUC (95% CI)	Cut-Off	Sensitivity	Specificity	*p*-Value	Global C-Index (95% CI)
HALLMOUNT	0.86 (0.82–0.91)	≥2	81.4%	74.2%	<0.001	0.80 (0.73–0.87)	≥3	79.6%	69.1%	0.002	0.78 (0.75–0.81)
NLR	0.59 (0.52–0.66)	≥3.1	61.3%	55.0%	0.12	0.54 (0.43–0.65)	≥3.4	57.8%	48.2%	0.20	0.55 (0.50–0.60)
CAR	0.84 (0.79–0.89)	≥0.4	83.0%	71.5%	<0.001	0.85 (0.77–0.92)	≥0.5	82.4%	74.8%	<0.001	0.77 (0.73–0.80)
PIV	0.86 (0.82–0.91)	≥350	85.7%	72.2%	<0.001	0.79 (0.72–0.86)	≥425	80.2%	70.4%	<0.001	0.77 (0.74–0.80)

**Table 5:** Univariate regression model of OS and PFS in patients with grade4 adult-type diffuse gliomas.

Univariate	Overall Survival			Progression-Free Survival		
	HR	95% CI for HR	*p*	HR	95% CI for HR	*p*
Age	1.55	1.17–2.03	0.002	1.39	1.06–1.83	0.019
Sex	0.93	0.70–1.23	0.600	0.97	0.74–1.28	0.833
Comorbidity	1.09	0.82–1.44	0.565	1.05	0.79–1.39	0.728
Smoking	1.17	0.88–1.56	0.288	1.06	0.79–1.41	0.715
ECOG PS	2.44	1.73–3.45	<0.001	2.32	1.64–3.26	<0.001
Lateralization	1.02	0.78–1.34	0.882	1.05	0.80–1.38	0.719
Tumor localization	1.12	0.98–2.11	0.511	1.95	1.08–2.16	0.410
Tumor focality	1.77	1.27–2.46	0.001	1.64	1.18–2.27	0.003
Tumor size	1.26	0.95–1.68	0.111	1.15	0.86–1.52	0.354
IDH mutation	3.43	2.36–4.97	<0.001	2.90	2.00–4.22	<0.001
ATRX loss	0.67	0.50–0.90	0.008	0.69	0.51–0.93	0.014
MGMT promoter methylation	0.76	0.64–0.92	0.003	0.76	0.64–0.91	0.003
The type of surgical resection ^µ^	0.55	0.42–0.73	<0.001	0.50	0.38–0.66	<0.001
Adjuvant radiotherapy	0.12	0.07–0.20	<0.001	0.14	0.09–0.24	<0.001
Adjuvant chemotherapy	0.14	0.09–0.20	<0.001	0.19	0.13–0.27	<0.001
Treatment response ^#^	0.75	0.63–0.90	0.001	0.88	0.74–1.05	0.153
NLR	1.50	1.12–2.03	0.007	1.52	1.12–2.05	0.007
CAR	3.65	2.71–4.91	<0.001	3.51	2.60–4.73	<0.001
PIV	3.02	2.26–4.04	<0.001	2.55	1.92–3.39	<0.001
HALLMOUNT	6.36	4.42–9.15	<0.001	4.89	3.48–6.88	<0.001

Abbreviations: ECOG PS, Eastern Cooperative Oncology Group Performance Status; IDH, isocitrate dehydrogenase; ATRX, Alpha thalassemia/mental retardation syndrome X-linked; MGMT, O6-methylguanine-DNA methyltransferase; ^µ^, Non-GTR, cases where only partial resection was achieved; cases involving biopsy alone were excluded; GTR, gross total resection; # (based on the findings from the initial response assessment conducted at week 12 following radiotherapy according to RANO 2.0) [28]; CR, complete response; PR, partial response; MR, minor response; SD, stable disease; PD, progressive disease; NLR, neutrophil-to-lymphocyte ratio; CAR, C-reactive protein-to-albumin ratio; PIV, pan-immune–inflammation value.

**Table 6:** Multivariate regression model of OS and PFS in patients with grade 4 adult-type diffuse gliomas.

Multivariate	Overall Survival			Progression-Free Survival		
	HR	95% CI for HR	*p*	HR	95% CI for HR	*p*
Age	1.04	(0.77–1.40)	0.815	1.12	(0.83–1.50)	0.472
ECOG PS	0.95	(0.65–1.39)	0.787	0.92	(0.62–1.37)	0.687
Tumor focality	1.63	(1.15–2.30)	0.006	–	–	–
IDH mutation	2.38	(1.50–3.77)	<0.001	1.92	(1.21–3.04)	0.006
ATRX loss	1.36	(0.96–1.91)	0.081	1.33	(0.94–1.89)	0.105
MGMT promoter methylation	0.92	(0.77–1.10)	0.364	0.87	(0.73–1.04)	0.121
The type of surgical resection ^µ^	0.63	(0.47–0.86)	0.003	0.60	(0.44–0.81)	0.001
Adjuvant radiotherapy	0.21	(0.12–0.38)	<0.001	0.26	(0.15–0.45)	<0.001
Adjuvan chemotherapy	0.51	(0.31–0.86)	0.010	0.58	(0.35–0.96)	0.033
Treatment response ^#^	0.99	(0.81–1.21)	0.923	1.25	(1.03–1.52)	0.022
NLR	1.44	(1.04–1.99)	0.028	1.28	(0.93–1.77)	0.135
CAR	2.47	(1.76–3.47)	<0.001	2.44	(1.74–3.42)	<0.001
PIV	1.99	(1.39–2.86)	<0.001	1.80	(1.28–2.54)	0.001
HALLMOUNT	2.78	(1.79–4.30)	<0.001	2.76	(1.82–4.19)	<0.001

Abbreviations: ECOG PS, Eastern Cooperative Oncology Group Performance Status; IDH, isocitrate dehydrogenase; ATRX, Alpha thalassemia/mental retardation syndrome X-linked; MGMT, O6-methylguanine-DNA methyltransferase; ^µ^, Non- GTR, cases where only partial resection was achieved; cases involving biopsy alone were excluded; GTR, Gross total resection; # (based on the findings from the initial response assessment conducted at week 12 following radiotherapy according to RANO 2.0) [28]; CR, complete response; PR, partial response; MR, minor response; SD, stable disease; PD, progressive disease; NLR, neutrophil-to-lymphocyte ratio; CAR, C-reactive protein-to-albumin ratio; PIV, Pan-immune–inflammation value.

## Data Availability

Access to the datasets analyzed in this study may be granted by the corresponding author on reasonable request, subject to approval by the Clinical Oncology Department of the University of Health Sciences Antalya Education and Research Hospital.

## Abbreviations

The following abbreviations are used in this manuscript:

ATRX	Alpha Thalassemia/Mental Retardation Syndrome X-linked
CNS	Central Nervous System
ECOG	Eastern Cooperative Oncology Group
GTR	Gross Total Resection
IDH	Isocitrate Dehydrogenase
LDH	Lactate Dehydrogenase
MGMT	O6-Methylguanine-DNA Methyltransferase
NCCN	National Comprehensive Cancer Network
NLRP3	NOD-, LRR-, and Pyrin Domain-containing Protein 3
OS	Overall Survival
PDGF	Platelet-derived Growth Factor
PFS	Progression-free Survival
RANO	Response Assessment in Neuro-Oncology
RT	Radiotherapy
TAMs	Tumor Associated Macrophages
TERT	Telomerase Reverse Transcriptase
TGF-β	Transforming Growth Factor-β
TMZ	Temozolomide
VEGF	Vascular Endothelial Growth Factor
WHO	World Health Organization
